# A Metabolomics Pilot Study on Desmoid Tumors and Novel Drug Candidates

**DOI:** 10.1038/s41598-017-18921-7

**Published:** 2018-01-12

**Authors:** Kelly A. Mercier, Mushriq Al-Jazrawe, Raymond Poon, Zachery Acuff, Benjamin Alman

**Affiliations:** 1 RTI International, Research Triangle Park, Durham, NC 27709 USA; 2 Developmental & Stem Cell Biology Program, Hospital for Sick Children, Toronto, ON Canada; 3 Laboratory Medicine & Pathobiology, University of Toronto, Toronto, ON Canada; 4 Department of Orthopaedics, Duke University, Durham, NC 27704 USA

## Abstract

Desmoid tumors (aggressive fibromatosis) are locally invasive soft tissue tumors that lack the ability to metastasize. There are no directed therapies or standard treatment plan, and chemotherapeutics, radiation, and surgery often have temporary effects. The majority of desmoid tumors are related to T41A and S45F mutations of the beta-catenin encoding gene (*CTNNB1*). Using broad spectrum metabolomics, differences were investigated between paired normal fibroblast and desmoid tumor cells from affected patients. There were differences identified, also, in the metabolomics profiles associated with the two beta-catenin mutations, T41A and S45F. Ongoing drug screening has identified currently available compounds which inhibited desmoid tumor cellular growth by more than 50% but did not affect normal fibroblast proliferation. Two drugs were investigated in this study, and Dasatinib and FAK Inhibitor 14 treatments resulted in unique metabolomics profiles for the normal fibroblast and desmoid tumor cells, in addition to the T41A and S45F. The biochemical pathways that differentiated the cell lines were aminoacyl-tRNA biosynthesis in mitochondria and cytoplasm and signal transduction amino acid-dependent mTORC1 activation. This study provides preliminary understanding of the metabolic differences of paired normal and desmoid tumors cells, their response to desmoid tumor therapeutics, and new pathways to target for therapy.

## Introduction

Desmoid tumors (aggressive fibromatosis) are locally invasive soft tissue tumors that account for 3% of soft tissue sarcomas^1^. Current treatments include, surgery, and radiation^2^, but treatments frequently have temporary results. For instance, between 23–50% patients have recurrence after surgical resection^3,4^. The first line of treatment is often watch-and-wait because of this probability of recurrence and lack of targeted therapeutics^5,6^.

Mutations of the beta-catenin encoding gene (*CTNNB1*) were identified with approximately 85% of sporadic tumors by Sanger sequencing, previously^7^, but next-generation sequencing estimates that these mutations may account for 90–95% of desmoid tumor cases^8^. Adenomatous polyposis coli (*APC*) gene mutations has been linked to the genetic disorder, familial adenomatous polyposis (FAP), in which at least 25% of patients develop desmoid tumors. Both of these genes are involved in the Wnt signaling pathway, which directs cell proliferation and adhesion, cell polarity, and cell fate determination^9^. Two mutations of the beta-catenin (T41A and S45F) account for 70% of the sporadic tumor formations^10^, and the S45F mutation has been correlated to an increase likelihood of local recurrence^10^. Additionally, in a small investigation, the S45F genotype has shown poor therapeutic response with the clooxygenase-2 (COX-2) selective inhibitor, Meloxicam^11^. Besides the genotyping data, additional immunohistochemical analysis yielded no definitive results among typical tumor and oncology markers^12^. Commercially available drug libraries can be used to screen compounds targeting desmoid tumor cells, and an ongoing screening procedure is identifying compounds that inhibited desmoid cellular growth but not normal fibroblast viability^13^. All of these compounds are either approved for use in patients or have the potential to be rapidly developed for patient care. Two compounds found in this ongoing screening, Dasatinib and FAK Inhibitor 14, target proteins that directly interact with the beta-catenin upstream of the Wnt pathway. Dasatinib is Bcr-Abl and Src family tyrosine kinase inhibitor^14^, while FAK inhibitors target the intersecting integrin and receptor tyrosine kinase signal transduction pathways^15^.

In recent years, the ‘rediscovery’ of how critical metabolic processes regulate tumor etiology and progression is identifying new prognostic factors and therapeutic approaches in a variety of tumors. To the best of our knowledge, this approach has not been applied to beta-catenin driven mesenchymal tumors. The importance of these findings is the opportunity to leverage the divergences from normal cellular functions into targetable features for novel drug development. To this end, metabolomics has become a valuable scientific tool in deciphering differential metabolic patterns and the factors that drive them. Cell line models are essential for biomarker discovery research^16^, as are establishing clinically relevant cell-based assays for target identification. Metabolomics has been instrumental, for instance, in understanding differences in metabolomics profiles and perturbed pathways between primary cell lines associated with triple negative breast cancer^17^, ovarian cancer, and colon cancer^18^. Additionally, it is possible to utilize metabolomics to determine the mechanism of action of novel therapeutics. One recent Nature Communications article thoroughly described how broad spectrum metabolomics is utilized when evaluating suspension and adherent cell lines in response to known kinase inhibitors^19^.

The goal of this project was to understand the underlying metabolomic differences in paired normal fibroblast and desmoid cells from affected patients and normal fibroblast cells from unaffected patients. Previous breast cancer research has indicated that it is important to understand the differences between normal tissue from affected and unaffected patients^20,21^. Moreover, the metabolic response to two novel desmoid drugs identified by the Desmoid Collaboration for a Cure was investigated. Broad spectrum metabolomics has not been applied to desmoid tumor research, and metabolic profiles differences in between desmoid tumor cells, normal fibroblast cells, and unaffected normal fibroblast cells were found readily. Additionally, there were differences identified in the metabolomics profiles associated with the two beta-catenin mutations, T41A and S45F, and in their response to two desmoid therapeutics, Dasatinib and FAK Inhibitor 14, identified from a drug screen. These data reflect differences in desmoid tumor biology, and the metabolic response to two chemotherapeutics provides important information for understanding how these effective therapies for desmoid tumors modify biological pathways.

## Results

### Metabolomics profiles distinguishes untreated cell lines

**Figure 1:**
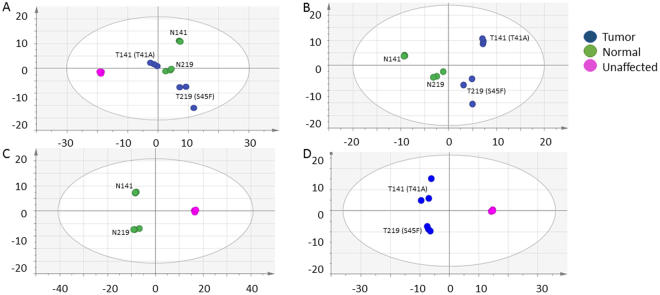
Multivariate analysis comparing untreated desmoid, normal, and unaffected. (**A**) In the unsupervised analysis, the 141 (T41A) and 219 (S45F) cell lines from affected subjects separate from the unaffected cell line from ATCC on the primary axis [5 components, R2X = 0.79, Q2 (cum) = 0.95]. (**B**) The supervised analysis of tumor and normal fibroblast [1 predictive + 1 orthogonal components, R2X = 0.71, R2Y = 0.82, Q2 (cum) = 0.71]. (**C**) The supervised analysis of normal and unaffected [1 predictive + 1 orthogonal components R2X = 0.97, R2Y = 1.0, Q2 (cum) = 1.0]. (**D**) The supervised analysis of tumor and unaffected (D) [1 predictive + 1 orthogonal components, R2X = 0.89, R2Y = 0.993, Q2 (cum) = 0.95].

To understand the differences between desmoid tumor, normal fibroblast, and unaffected fibroblast, broad spectrum metabolomics was used to investigate the untreated cell lines. Unsupervised multivariate analysis (PCA) and supervised analysis (OPLS-DA) differentiated the untreated cell lines. As shown in Fig. 1A, the PCA shows the cell types between unaffected and affected, in addition to tumor and normal. The unaffected cell line was separated from the affected cell lines on the 1^st^ component of the PCA, whereas the matched desmoid tumor and normal cell lines were along the 2^nd^ PCA component. Supervised analysis (OPLS-DA) was used to distinguish tumor from normal (Fig. 1B), normal from unaffected (Fig. 1C), and tumor from unaffected (Fig. 1D). In these comparisons, it is clear that the metabolomic profiles of the two tumor and paired normal cell lines differ from the unaffected. While the predictive 1st component in these three supervised analysis differentiated the cell type, the orthogonal component is dominated, again, by the genotypes. The NMR bins associated with defining the differences between the cell lines is listed in Table 1, which included library matched metabolites which were found to be VIP ≥ 1.0, p < 0.1, or FC > 2. It was found that the lipids were greatest in the unaffected cell line compared to the affected. The desmoid tumor cell lines had higher lipids than their matched normal. The bins with identified amino acids (alanine, leucine, isoleucine, valine, glutamate, glutamine, serine, threonine, and tyrosine) experienced a higher fold change in the unaffected cell line compared to the affected cell lines. Additionally, these same amino acids were noted also to be higher fold change in the tumor compared to the matched normal cells. The exception, however, was aspartate and glycine, where the bins associated with this amino acid had a higher fold change in the normal cells than the tumor cells. It was also interesting that asparagine and phenylalanine were differentiated in the affected and unaffected, while only methionine differentiated in the normal and unaffected binned NMR profiles. Dimethylamine was found to be lowest in the tumor cell lines. Metabolites were semi-quantified, and Table 2 shows the metabolites that were found to be p < 0.1 or FC > 2. Interestingly, glucose 1-phosphate was significantly higher in both affected cell lines compared to the unaffected cell line, with the normal and tumor median concentrations 210 and 140 µM, respectively.

**Table 1:** The library matched NMR bins that differentiate the untreated desmoid, tumor, and unaffected cell lines where the p values were calculated with the Wilcoxon Rank Sum Test (normal vs tumor) and exact Wilcoxon Rank Sum Test (tumor vs unaffected and normal vs unaffected) (VIP ≥ 1.0, p < 0.1, or FC > 2).

Library-Matched Metabolite	Chemical Shift	Tumor vs Normal			Normal vs Unaffected			Tumor vs Unaffected			Cell Line Difference
		VIP	p-value*	FC**	VIP	p-value*	FC**	VIP	p-value*	FC**	
Lipids	[0.50.. 0.52]				0.4	0.024	−2.0	0.5	0.024	−2.6	Unaffected > Normal > Tumor
Lipids	[0.52.. 0.55]				0.4	0.024	−1.8	0.5	0.048	−2.0	Unaffected > Normal > Tumor
Lipids	[0.55.. 0.57]				0.3	0.024	−1.4	0.4	0.048	−1.3	Unaffected > Tumor > Normal
Lipids	[0.57.. 0.59]				0.4	0.024	−1.5	0.5	0.024	−1.7	Unaffected > Normal > Tumor
Lipids	[0.59.. 0.62]				0.4	0.024	−1.5	0.5	0.024	−1.7	Unaffected > Normal > Tumor
Lipids	[0.62.. 0.64]				0.4	0.024	−1.3	0.5	0.024	−1.3	Unaffected > Tumor > Normal
Lipids	[0.64.. 0.66]				0.4	0.024	−1.4	0.5	0.024	−1.5	Unaffected > Normal > Tumor
Lipids	[0.66.. 0.68]				0.5	0.024	−1.5	0.6	0.024	−1.5	Unaffected > Normal > Tumor
Lipids	[0.68.. 0.70]				0.5	0.024	−1.4	0.6	0.024	−1.4	Unaffected > Normal > Tumor
Lipids	[0.70.. 0.72]				0.5	0.024	−1.4	0.6	0.024	−1.4	Unaffected > Normal > Tumor
Lipids	[0.72.. 0.74]				0.5	0.024	−1.4	0.6	0.024	−1.4	Unaffected > Tumor > Normal
Lipids	[0.74.. 0.77]				0.6	0.024	−1.3	0.6	0.024	−1.3	Unaffected > Tumor > Normal
Lipids	[0.77.. 0.79]				0.6	0.024	−1.3	0.6	0.024	−1.3	Unaffected > Normal > Tumor
Lipids	[0.79.. 0.81]				0.5	0.048	−1.3	0.6	0.024	−1.3	Unaffected > Normal > Tumor
Lipids	[0.81.. 0.83]				0.5	0.095	−1.2	0.5	0.048	−1.3	Unaffected > Normal > Tumor
Lipids	[0.83.. 0.89]				1.1	0.024	−1.2	1.0	0.548	−1.1	Unaffected > Tumor > Normal
Lipids| Glycocholate	[0.89.. 0.91]				0.7	0.024	−1.2				Unaffected > Normal
	[0.91.. 0.97]	2.3	0.255	1.1	2.2	0.024	−1.4	2.1	0.024	−1.3	Unaffected > Tumor > Normal
Valine | Isoleucine	[0.97.. 1.02]	1.6	0.397	1.1	1.4	0.024	−1.3	1.3	0.024	−1.3	Unaffected > Tumor > Normal
Valine	[1.02.. 1.07]	1.5	0.938	−1.0	1.3	0.024	−1.4	1.5	0.024	−1.4	Unaffected > Normal > Tumor
Unknown	[1.07.. 1.10]				0.4	0.024	−1.2	0.6	0.024	−1.3	Unaffected > Normal > Tumor
Unknown	[1.10.. 1.12]				0.4	0.024	−1.2	0.5	0.024	−1.3	Unaffected > Normal > Tumor
Unknown	[1.12.. 1.14]				0.4	0.024	−1.2	0.5	0.024	−1.2	Unaffected > Normal > Tumor
Lipids	[1.14.. 1.16]				0.4	0.024	−1.2	0.5	0.024	−1.2	Unaffected > Normal > Tumor
Lipids	[1.16.. 1.18]				0.4	0.095	−1.1	0.4	0.024	−1.2	Unaffected > Normal > Tumor
Isoleucine | 3-Hydroxyisovalerate	[1.23.. 1.27]							0.8	0.024	−1.3	Unaffected > Tumor
Isoleucine	[1.27.. 1.30]							0.7	0.024	−1.3	Unaffected > Tumor
Lactate | Threonine	[1.30.. 1.34]	1.7	0.320	1.1	1.8	0.024	−1.4	1.7	0.024	−1.2	Unaffected > Tumor > Normal
Lipids	[1.34.. 1.36]				0.5	0.024	−1.2	0.4	0.024	−1.2	Unaffected > Tumor > Normal
Lipids	[1.36.. 1.39]	0.5	0.093	1.1	0.6	0.024	−1.2				Unaffected > Tumor > Normal
Lipids	[1.39.. 1.44]				1.0	0.024	−1.2	0.8	0.024	−1.1	Unaffected > Tumor > Normal
Alanine| Lipids	[1.44.. 1.49]	2.0	0.397	1.0	1.8	0.024	−1.4	1.8	0.024	−1.3	Unaffected > Tumor > Normal
Lipids	[1.49.. 1.55]	1.2	0.397	1.0	1.2	0.024	−1.4	1.2	0.024	−1.4	Unaffected > Tumor > Normal
Lipids	[1.55.. 1.57]				0.6	0.024	−1.3	0.6	0.024	−1.2	Unaffected > Tumor > Normal
Lipids	[1.57.. 1.59]				0.5	0.024	−1.3	0.5	0.024	−1.2	Unaffected > Tumor > Normal
Lipids	[1.59.. 1.61]				0.5	0.024	−1.2	0.4	0.048	−1.2	Unaffected > Tumor > Normal
Leucine	[1.61.. 1.64]				0.7	0.024	−1.3	0.6	0.024	−1.2	Unaffected > Tumor > Normal
Leucine	[1.64.. 1.66]				0.7	0.024	−1.3	0.7	0.024	−1.2	Unaffected > Tumor > Normal
Leucine	[1.66.. 1.68]	0.7	0.054	1.1	0.8	0.024	−1.3	0.7	0.024	−1.2	Unaffected > Tumor > Normal
Leucine | Lysine| Lipids	[1.68.. 1.73]	2.1	0.320	1.1	1.9	0.024	−1.4	1.9	0.024	−1.4	Unaffected > Tumor > Normal
Leucine | Lysine| Lipids	[1.73.. 1.79]	1.4	0.586	1.1	1.4	0.024	−1.4	1.4	0.024	−1.3	Unaffected > Tumor > Normal
Lipids	[1.79.. 1.84]				1.0	0.024	−1.3	1.0	0.024	−1.2	Unaffected > Tumor > Normal
Lipids	[1.84.. 1.86]				0.7	0.024	−1.3	0.6	0.024	−1.2	Unaffected > Tumor > Normal
Lysine| Lipids	[1.86.. 1.88]				0.7	0.024	−1.4	0.7	0.024	−1.2	Unaffected > Tumor > Normal
Arginine | Lysine	[1.88.. 1.94]	1.9	0.397	1.1	1.7	0.024	−1.4	1.8	0.024	−1.4	Unaffected > Tumor > Normal
Lysine| Proline	[1.94.. 1.96]				0.8	0.024	−1.4	0.7	0.024	−1.2	Unaffected > Tumor > Normal
Proline	[1.96.. 1.99]	1.2	0.093	1.2	1.1	0.024	−1.4	1.0	0.024	−1.2	Unaffected > Tumor > Normal
Proline	[1.99.. 2.01]	1.1	0.093	1.1	1.0	0.024	−1.4	0.9	0.024	−1.2	Unaffected > Tumor > Normal
Pyroglutamate | Glutamate | Proline	[2.01.. 2.07]	1.6	0.093	1.1	1.7	0.024	−1.2	1.3	0.548	−1.1	Unaffected > Tumor > Normal
Glutamate	[2.07.. 2.09]	0.8	0.054	1.1	0.9	0.024	−1.3	0.7	0.024	−1.2	Unaffected > Tumor > Normal
Glutamate | Glutamine	[2.09.. 2.15]	1.7	0.938	1.0							Tumor > Normal
Glutamate | Glutamine | Glutathione	[2.15.. 2.19]				0.9	0.024	−1.2	0.9	0.024	−1.1	Unaffected > Tumor > Normal
Unknown	[2.19.. 2.21]				0.5	0.024	−1.1	0.5	0.024	−1.1	Unaffected > Tumor > Normal
Lipids	[2.21.. 2.24]				0.6	0.024	−1.2	0.6	0.024	−1.1	Unaffected > Tumor > Normal
Valine | Lipids	[2.24.. 2.30]	1.2	0.397	1.1	1.2	0.024	−1.3	1.2	0.024	−1.2	Unaffected > Tumor > Normal
Glutamate	[2.30.. 2.32]				0.7	0.024	−1.2	0.5	0.048	−1.1	Unaffected > Tumor > Normal
Glutamate | Proline	[2.32.. 2.37]	2.0	0.201	1.1	2.0	0.024	−1.2	1.5	0.381	−1.1	Unaffected > Tumor > Normal
Pyroglutamate | Glutamine	[2.37.. 2.40]				0.8	0.024	−1.3	0.8	0.024	−1.4	Unaffected > Normal > Tumor
Glutamine| Pyroglutamate	[2.40.. 2.42]	0.4	0.023	−1.2				0.6	0.024	−1.2	Unaffected > Normal > Tumor
Glutamine	[2.42.. 2.48]	1.7	0.040	−1.2	1.2	0.024	1.2				Normal > Tumor > Unaffected
Pyroglutamate| Glutathione	[2.48.. 2.51]							0.7	0.024	−1.3	Unaffected > Tumor
Glutathione	[2.51.. 2.57]				0.9	0.024	−1.3	1.0	0.095	−1.2	Unaffected > Tumor > Normal
Glutathione	[2.57.. 2.60]				0.6	0.024	−1.3	0.7	0.048	−1.2	Unaffected > Tumor > Normal
Unknown	[2.60.. 2.62]				0.3	0.024	−1.3	0.4	0.095	−1.3	Unaffected > Tumor > Normal
Methionine	[2.62.. 2.66]				0.5	0.024	1.1				Normal > Unaffected
Aspartate	[2.66.. 2.72]	0.9	0.017	−1.1	1.0	0.024	−1.3	1.3	0.024	−1.4	Unaffected > Normal > Tumor
Dimethylamine	[2.72.. 2.77]				0.5	0.024	−1.1	0.7	0.024	−1.2	Unaffected > Normal > Tumor
Aspartate	[2.77.. 2.80]	0.8	0.054	−1.1	0.8	0.024	−1.4	1.0	0.024	−1.6	Unaffected > Normal > Tumor
Aspartate	[2.80.. 2.83]	0.7	0.071	−1.1	0.7	0.024	−1.3	0.9	0.024	−1.5	Unaffected > Normal > Tumor
Asparagine	[2.83.. 2.87]				0.6	0.024	−1.5	0.7	0.095	−1.3	Unaffected > Tumor > Normal
Asparagine	[2.87.. 2.90]				0.6	0.024	−1.4	0.7	0.024	−1.4	Unaffected > Tumor > Normal
Tyramine	[2.90.. 2.92]				0.6	0.024	−1.7				Unaffected > Normal
Asparagine	[2.92.. 2.95]				0.9	0.024	−1.7				Unaffected > Normal
Asparagine	[2.95.. 2.97]				0.8	0.024	−1.4	0.6	0.024	−1.2	Unaffected > Tumor > Normal
Glutathione | Lysine| Lipids	[2.97.. 3.02]	1.0	0.121	1.1	1.1	0.024	−1.3	1.0	0.024	−1.2	Unaffected > Tumor > Normal
Creatine | Creatinine | Lysine| Tyrosine	[3.02.. 3.07]	1.7	0.397	1.1	1.7	0.024	−1.6	1.7	0.024	−1.4	Unaffected > Tumor > Normal
Lipids	[3.07.. 3.09]				0.5	0.024	−1.5	0.6	0.095	−1.2	Unaffected > Tumor > Normal
Phenylalanine	[3.09.. 3.13]				0.9	0.024	−1.3	0.9	0.024	−1.2	Unaffected > Tumor > Normal
Phenylalanine	[3.13.. 3.17]				0.7	0.024	−1.3	0.8	0.024	−1.2	Unaffected > Tumor > Normal
Tyrosine	[3.17.. 3.19]				0.6	0.024	−1.4	0.6	0.024	−1.1	Unaffected > Tumor > Normal
Choline | O-Phosphocholine | O-Acetylcholine | sn-Glycero-3-phosphocholine	[3.19.. 3.23]	2.0	0.017	1.3	1.8	0.024	−1.3	0.9	0.024	−1.0	Unaffected > Tumor > Normal
myo-Inositol | Glucose | Taurine	[3.23.. 3.29]	1.4	0.815	1.0	1.5	0.024	1.2	1.5	0.381	1.2	Tumor > Normal > Unaffected
myo-Inositol	[3.29.. 3.31]	1.2	0.017	−2.1	1.0	0.024	1.9				Normal > Unaffected > Tumor
Proline	[3.31.. 3.33]				0.6	0.024	−1.4	0.8	0.024	−1.6	Unaffected > Normal > Tumor
Methanol | Proline	[3.33.. 3.36]				0.9	0.024	−1.3	0.9	0.024	−1.2	Unaffected > Tumor > Normal
Glucose|Glucose-1-phosphate	[3.36.. 3.39]	1.4	0.815	−1.0	1.5	0.024	5.5	1.6	0.024	5.3	Normal > Tumor > Unaffected
Glucose|Glucose-1-phosphate | Taurine	[3.39.. 3.44]	2.3	0.017	1.2	2.8	0.024	2.1	3.5	0.024	2.5	Tumor > Normal > Unaffected
Glucose	[3.44.. 3.46]				0.8	0.024	1.8				Normal > Unaffected
Glucose|Glucose-1-phosphate	[3.46.. 3.51]	3.0	0.815	1.1	3.3	0.024	4.0	3.7	0.024	4.3	Tumor > Normal > Unaffected
Glucose|myo-Inositol | Glycine	[3.51.. 3.57]	3.5	0.040	−1.7	3.1	0.024	2.0	1.7	1.000	1.2	Normal > Tumor > Unaffected
myo-Inositol| Threonine	[3.57.. 3.62]	2.7	0.040	−1.7	2.3	0.024	1.8	1.2	1.000	1.0	Normal > Tumor > Unaffected
Glycerol | Unknown	[3.62.. 3.68]	1.4	0.017	−1.3	1.4	0.048	1.3	0.5	0.048	−1.0	Normal > Unaffected > Tumor
Glucose	[3.68.. 3.70]							0.6	0.024	−1.1	Unaffected > Tumor
Glucose	[3.70.. 3.72]				1.0	0.024	1.6				Normal > Unaffected
Glucose-1-phosphate |Glutamate	[3.72.. 3.74]	1.8	1.000	1.0	2.0	0.024	2.6	2.2	0.024	2.6	Tumor > Normal > Unaffected
Glucose-1-phosphate |Glutamate |Alanine	[3.74.. 3.80]	3.3	1.000	1.1	3.4	0.024	1.4	4.0	0.024	1.5	Tumor > Normal > Unaffected
Glucose-1-phosphate| Serine	[3.83.. 3.89]	2.4	1.000	1.0	2.8	0.024	1.7	3.2	0.024	1.8	Tumor > Normal > Unaffected
Glucose-1-phosphate | Creatine	[3.89.. 3.93]	2.7	1.000	1.1	2.9	0.024	1.9	3.3	0.024	2.1	Tumor > Normal > Unaffected
Serine | Unknown	[3.93.. 3.97]	1.9	0.071	1.1	1.7	0.024	−1.5	1.5	0.024	−1.3	Unaffected > Tumor > Normal
Serine | Unknown	[3.97.. 4.02]	2.4	0.586	1.1	1.9	0.024	−1.6	1.8	0.167	−1.4	Unaffected > Tumor > Normal
myo-Inositol| Choline	[4.02.. 4.08]	1.9	0.040	−1.5	1.6	0.024	1.4	1.0	1.000	−1.1	Normal > Unaffected > Tumor
Ribose| Lactate	[4.08.. 4.11]				0.8	0.024	−1.2	0.8	0.024	−1.2	Unaffected > Tumor > Normal
Lactate| O-Phosphocholine| Proline| Uridine	[4.11.. 4.17]	0.9	0.023	1.1	1.1	0.024	−1.2	0.6	0.095	−1.1	Unaffected > Tumor > Normal
O-Phosphocholine | Pyroglutamate	[4.17.. 4.19]				0.6	0.024	−1.3	0.6	0.024	−1.4	Unaffected > Normal > Tumor
Threonine	[4.22.. 4.28]				0.9	0.024	−1.2	0.8	0.048	−1.2	Unaffected > Tumor > Normal
Lipids	[4.28.. 4.34]	1.2	0.156	1.1	1.2	0.024	−1.4	1.1	0.024	−1.3	Unaffected > Tumor > Normal
1-Methylnicotinamide	[4.45.. 4.50]				0.7	0.024	−1.3	0.6	0.024	−1.2	Unaffected > Tumor > Normal
Unknown	[4.50.. 4.54]				0.5	0.024	−1.5	0.6	0.024	−1.7	Unaffected > Normal > Tumor
Glutathione	[4.54.. 4.59]				0.7	0.024	−1.6				Unaffected > Normal
Unknown	[4.59.. 4.63]				0.4	0.024	−1.5				Unaffected > Normal
Glucose	[4.63.. 4.68]				0.6	0.048	1.6				Normal > Unaffected
Glucose	[5.20.. 5.26]				0.7	0.048	2.0				Normal > Unaffected
Unknown	[5.32.. 5.37]	0.3	0.317	−2.1				0.4	0.095	−2.6	Unaffected > Normal > Tumor
Unknown	[5.37.. 5.42]	0.5	0.053	−8.9	0.6	1.000	2.3	0.4	0.048	−4.0	Normal > Unaffected > Tumor
Glucose-1-phosphate	[5.42.. 5.48]	2.7	0.697	1.2	2.9	0.024	72.0	3.3	0.024	83.7	Tumor > Normal > Unaffected
Unknown	[5.48.. 5.53]	0.2	0.076	−6.9	0.2	0.095					Normal > Tumor > Unaffected
Uracil	[5.76.. 5.82]				0.6	0.024	−8.7	0.7	0.012	0.0	Unaffected > Normal > Tumor
Uridine	[5.88.. 5.94]	1.2	1.000	1.0	0.9	0.024	22.0	1.0	0.024	22.4	Tumor > Normal > Unaffected
Tyrosine	[6.87.. 6.93]				0.6	0.024	−1.3	0.6	0.024	−1.2	Unaffected > Tumor > Normal
Unknown	[7.06.. 7.08]				0.3	0.048	−1.5	0.3	0.024	−1.6	Unaffected > Normal > Tumor
p-Methylhistidine	[7.08.. 7.14]				0.4	0.024	−1.2	0.3	0.095	−1.1	Unaffected > Tumor > Normal
Tyrosine	[7.16.. 7.22]				0.7	0.024	−1.3				Unaffected > Normal
Unknown	[7.22.. 7.27]				0.3	0.024	−1.1				Unaffected > Normal
Phenylalanine	[7.27.. 7.29]				0.3	0.024	−1.4				Unaffected > Normal
Phenylalanine	[7.29.. 7.34]				0.8	0.024	−1.4				Unaffected > Normal
Phenylalanine	[7.34.. 7.39]				0.6	0.024	−1.5	0.6	0.095	−1.4	Unaffected > Tumor > Normal
Unknown	[7.39.. 7.45]				0.8	0.024	−1.6	0.7	0.024	−1.4	Unaffected > Tumor > Normal
Uracil	[7.45.. 7.51]				0.5	0.024	−3.8	0.4	0.155	−2.5	Unaffected > Tumor > Normal
Unknown	[7.51.. 7.57]				0.8	0.024	−2.8	0.9	0.024	−3.7	Unaffected > Normal > Tumor
Unknown	[7.71.. 7.76]	0.3	0.317	−3.2	0.4	0.048	−3.3	0.5	0.036	−10.6	Unaffected > Normal > Tumor
Uridine	[7.84.. 7.89]	1.0	0.697	−1.1	0.7	0.024	3.4	0.7	0.024	3.2	Normal > Tumor > Unaffected
p-Methylhistidine	[7.91.. 7.97]				0.3	0.095	−1.4	0.4	0.095	−1.4	Unaffected > Tumor > Normal
Unknown	[8.33.. 8.38]	0.1	0.070	−2.5				0.3	0.155	−4.8	Unaffected > Normal > Tumor
Unknown	[8.44.. 8.47]	0.1	0.076	−19.5				0.4	0.024	−20.2	Unaffected > Normal > Tumor
1-Methylnicotinamide	[8.92.. 8.95]				0.2	0.048	−3.4	0.3	0.024	−6.1	Unaffected > Normal > Tumor
1-Methylnicotinamide	[9.27.. 9.31]							0.3	0.095	−1.5	Unaffected > Tumor

**Table 2:** Semi-quantitated metabolites that were found to be statistically different based on the median values for untreated tumor, normal and unaffected cells. The p values were calculated with the Wilcoxon Rank Sum Test (normal vs tumor) and exact Wilcoxon Rank Sum Test (tumor vs unaffected and normal vs unaffected) (p < 0.1, or FC > 2).

Metabolite	Tumor vs Normal		Normal vs Unaffected		Tumor vs Unaffected		Cell Line Difference
	p-value*	FC**	p-value*	FC**	p-value*	FC**	
Aspartate					0.048	−1.7	Unaffected > Tumor
Dimethylamine	0.023	−1.3			0.095	−1.4	Tumor < (Normal| Unaffected)
Glucose-1-phosphate	0.04	−1.5	0.024	55.5	0.024	36	Normal > Tumor > Unaffected
Glutamine	0.017	−1.5	0.024	1.4			Normal > (Tumor| Unaffected)
Leucine					0.024	−2	Unaffected > Tumor
Phenylalanine					0.095	−1.7	Unaffected > Tumor
Pyroglutamate	0.023	−1.4	0.095	−1.3	0.024	−1.9	Unaffected > Normal > Tumor
Tyrosine					0.095	−1.4	Unaffected > Tumor

Metabolites identified were incorporated in the pathway enrichment analysis which was performed using the knowledge-based canonical pathways and endogenous metabolic pathways in the MetaCore module of the GeneGo software. Rankings of the canonical pathways were based on hypergeometric *p*-values and false discovery rate (FDR) being < 0.05. The top hits included aminoacyl-tRNA biosynthesis in mitochondria and cytoplasm and signal transduction amino acid-dependent mTORC1 activation (Table 3).

**Table 3:** Top ten statistically significant pathway maps created using GeneGo enrichment analysis based on library-matched metabolites the tumor, normal, and unaffected cell lines and are ranked using hypergeometric *p*-values and false discovery rate (FDR).

		Tumor vs Normal		Tumor vs Unaffected		Normal vs Unaffected	
		p-value	FDR	p-value	FDR	p-value	FDR
1	Aminoacyl-tRNA biosynthesis in mitochondrion	7.62E-19	1.39E-16	1.09E-21	2.04E-19	4.82E-23	9.11E-21
2	Aminoacyl-tRNA biosynthesis in cytoplasm	1.11E-17	7.83E-16	2.47E-20	1.81E-18	1.34E-21	1.01E-19
3	Aminoacyl-tRNA biosynthesis in cytoplasm/ Rodent version	1.29E-17	7.83E-16	2.90E-20	1.81E-18	1.61E-21	1.01E-19
4	Signal transduction_Amino acid-dependent mTORC1 activation	5.18E-16	2.36E-14	2.24E-15	1.05E-13	5.00E-15	2.36E-13
5	Nociception_Pro-nociceptive action of Nociceptin in spinal cord at low doses	1.66E-06	5.04E-05	2.78E-06	1.04E-04	3.68E-06	1.39E-04
6	Glycine, serine, cysteine and threonine metabolism	1.74E-06	5.04E-05	3.51E-06	1.05E-04	5.16E-06	1.55E-04
7	Glycine, serine, cysteine and threonine metabolism/ Rodent version	1.94E-06	5.04E-05	3.91E-06	1.05E-04	5.75E-06	1.55E-04
8	Nicotine signaling in cholinergic neurons	4.03E-06	9.16E-05	6.69E-06	1.56E-04	8.85E-06	2.09E-04
9	Galactose metabolism	1.00E-05	2.02E-04	1.66E-05	3.44E-04	2.18E-05	4.59E-04
10	Leucine, isoleucine and valine metabolism	5.37E-05	9.78E-04	8.02E-05	1.50E-03	1.00E-04	1.89E-03

To further investigate the differences between the two genotypes, a supervised analysis exhibited a well-defined separation (Supplemental Fig. 1), and Supplemental Tables 1 and 2 list the metabolites identified and semi-quantified that were significantly different. The metabolites and pathways that differentiated the normal and tumor cells lines were different from the two tumor cell lines. Two metabolites were noted to have high fold change differences between the genotypes in the binned data. The bins associated with 1-methylnicotinamide was between 2.1–4.5 fold higher in S45F; additionally, the bin identified as p-methylhistidine was 4.5 fold higher in the S45F cell line. 1-methylnicotinamide is involved in NAD metabolism, while p-methylhistidine is involved in beta-alanine and histadine metabolism. The semi-quantified metabolites were all greater in the T219 cell line.

### Treatment of cells with Dasatinib and FAK Inhibitor 14 alters metabotype of all cell lines

**Figure 2:**
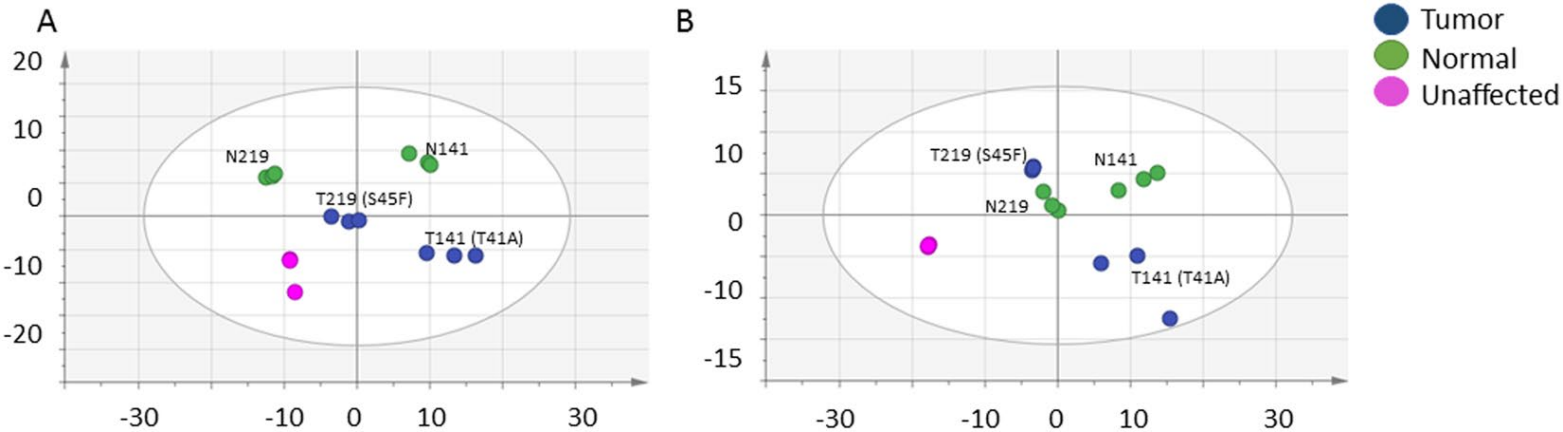
The unsupervised multivariate analysis (PCA) of desmoid, normal, and unaffected cell lines in the presence of Dasatinib (**A**) and FAK Inhibitor 14 (**B**). The PCA in the presence of Dasatinib [6 components, R2X = 0.99, Q2 (cum) = 0.92]. The PCA in the presence of FAK Inhibitor 14 [7 components, R2X = 0.99, Q2 (cum) = 0.94].

The differences between the response to Dasatinib and FAK Inhibitor 14 by the desmoid tumor, normal, and unaffected cell lines were investigated with the unsupervised multivariate analysis (PCA), as show in Fig. 2A–B. The differentiation of the metabolomics profiles were affected and unaffected on the first component. The Dasatinib treated cells separated by tumor and normal on the 2^nd^ component (A); the FAK Inhibitor 14 cells did not (B). The supervised analysis of the Dasatinib and FAK Inhibitor 14 treated cells showed excellent differentiation between the treated and vehicle (Supplemental Fig. 2A–F), and Supplemental Table 3 and 4 list the metabolites identified and semi-quantified metabolites that distinguish the two treatments. In the Dasatinib treatment, it was observed that the lipid bins in the normal cell lines were lower in the vehicle, while there was no significant difference in the tumor cells lines. In contrast, there was a lipid increase in the tumor cells and a decrease in the normal cells when treated with the FAK Inhibitor 14. The bins identified as 1-methylnicotinamide were observed to be significantly different in the FAK Inhibitor 14 treatment in the tumor cell lines, while there was no difference in the Dasatinib treatment.

As was seen in the untreated comparison, the two genotypes differentiated in the treatment responses, and the supplemental Table 5 highlight the semi-quantitative therapeutic responses. Generally, the treatments caused the metabolites to increase in concentration in the T219 (S45F) cell line and decrease in the T141 (T41A) cell line. It was observed, however, that the Dasatinib treatment resulted in a 25.5-fold increase in glucose-1-phosphate in the T219 cell line, but no significant change in either treatment in the T141 cell line. There were similarities in the response for the normal and the T141 cells; both had decreases in glutathione and pyroglutamate, while there was a slight increase or no change in the T219 cells for these two, respectively. While there was a slight increase in uridine in the T219, Dasatinib treatment, there was an 8.8-fold decrease when treated with the FAK Inhibitor 14.

## Discussion

To better understand underlying desmoid tumor cell behavior, this pilot broad spectrum metabolomics project was used to investigate metabolomic differences in matched normal and desmoid tumor cells with unaffected fibroblast cells. These cells are derived from affected patients and may have been the only matched cell lines that exist to date for this rare tumor type. While a small study, this provides a unique opportunity to understand the differences between affected and unaffected fibroblast cells. The cells clearly separated by unsupervised and supervised multivariate analysis and semi-quantitative concentration by affected and unaffected, in addition to tumor, normal, and unaffected. Thus, the metabolomic profile of desmoid tumors and normal fibroblasts from the same patient differ. Most studies have evaluated affected and unaffected tissue; it is not unexpected that the normal and unaffected fibroblast cells differentiate^22^. Huang, Stern, and Zhao showed that the studying tumors micro-environments may be more informative for markers of genetic susceptibility and disease monitoring in breast cancer, kidney clear cell carcinoma, lung adenocarcinoma, liver cancer, lung squamous cell carcinoma, and head and neck cancer. These paired samples are rare; even in breast cancer research, only 105 paired samples have been identified and studied. The micro-environment is particularly relevant for desmoid tumors, which lack the ability to metastasize^7^.

The metabolites that were found to be significantly different in the normal, desmoid tumor, and unaffected (Tables 1 and 2) were represented in the aminoacyl-tRNA biosynthesis in mitochondria and cytoplasm (Table 3). In most cases, the amino acids and lipids were greatest in the unaffected cell lines, and the desmoid tumor cell lines had greater concentrations than normal. Differences in the amino acid concentrations may be an indicator that there is a dysfunction in aminoacyl-tRNA synthetases. The aminoacyl-tRNA synthetases (ARSs) are responsible for protein translation, catalyse the ligation of amino acids to their cognate tRNAs, and interact with various proteins with tumorigenesis implications^23,24^. Aspartate and glycine were two amino acids that were greater in the normal cells compared to the desmoid tumor. The aspartyl-tRNA synthetase (AspRD) is known to interact with the multisynthetase complex p43 (MSC^p43^), which in turns antiproliferative and proapoptotic signaling pathways related to FUSEbinding protein (FBP), Myc, TNF receptorassociated factor 2 (TRAF2) and p53^25^. Glycyl-tRNA synthetase (GRS) has been previously found to be overexpressed in breast cancer cell lines^26^, in addition to GRS being secreted to the serum in defense of ERK-activated tumorigenesis^27^. Another amino acid that differentiated the cell types was tyrosine. In addition to the normal ARS functions, Tyrosyl-tRNA synthetase (TyrRS) contains the tripeptide ELR (GluLeuArg) and the EMAPII domain^28^. The ELR peptide has been identified in lung cancer as a chemokine^29^. Additionally, the EMAPII domain inhibits integrin-dependent cell adhesion with the interaction of α5β1 integrin receptor on the surface of endothelial cells^28^. As previously noted, Wnt/beta-Catenin signaling also play a critical role in cell adhesion, and Yum *et al*. recently investigated the relationship between AIMP2, such as TyrRS, and Wnt/beta-catenin signaling in a murine model of intestinal homeostasis and tumorigenesis^29^. The AIMP2 was thusly found to regulate the Wnt/beta-catenin signaling in the intestinal tract; however, it is not clear from their results if or how the signaling cascade would be altered with the known desmoid tumor mutation of beta-catenin, T41A or S45F. ARS have been identified as drug targets, such as aspartyl-tRNA synthetase for Mycobacterium tuberculosis^30^, or glycyl-tRNA synthetase for Charcot-Marie-Tooth peripheral neuropathy^31^. Because it is possible to target one ARS verses the others using known binding motifs^32^, future screening experiments would target several ARS inhibitors against these cell lines to find most efficacious results.

Another differentiated pathway was signal transduction amino acid-dependent mTORC1 activation pathways. The mTOR complex 1(mTORC1) signaling is reduced dramatically in the absence of arginine and the branched chain amino acids^33^. The activity is recovered when the amino acids, particularly leucine, are added back into the reaction^34^. Leucyl-tRNA synthetase (LARS) was identified as sensor in the mTORC1 signaling, also independent of its translational function^35^. Furthermore, there was a clear difference in the glucose and glucose-1-phosphate concentrations in the normal, desmoid tumor, and unaffected cell lines. In an elegant set of experiments, Pusapati *et al*. found that the activation of mTORC1 signaling in solid tumors can avoid glycolysis block and ensure adequate energy for cellular survival^36^. The metabolomics results and these ARS and mTORC1 results indicate that further investigations are needed to further understand this pathways in desmoid tumorigenesis. mTORC1 has been shown to play a role in cell proliferation and tumor growth in transgenic animals carrying APC mutations similar to what is seen in desmoid tumor patients^37^. Sirolimus, also known as rapamycin, is an immunosuppressive agent and known mTOR inhibitor, and is currently being investigated in a pediatric desmoid tumor clinical trial (NCT01265030). The primary outcome of this trial is to determine if mTOR inhibition is beneficial for children and young adults with desmoid tumors. Future metabolomic studies will also investigate the response of the sirolimus in the cell lines.

The metabolomics profiles for the two genotypes (T41A and S45F) were also distinct and revealed 1-methylnicotinamide and p-methylhistidine as being significantly greater in the S45F cell line. 1-methylnicotinamide is involved in NAD metabolism. Like mTORC1 signaling, NAD metabolism has also been suggested as a vital metabolic rewiring for energy metabolism^38^ P-methylhistidine is involved in histidine metabolism, which has been noted previously as markers of patients with cachexia and pancreatic cancer^39^. p-methylhistadine is also known to bind histidine decarboxylase, where Masini *et al*. found that histamine concentration and histidine decarboxylase activity is correlated with colorectal cancer tumor stages and intratumor microvessel density^40^. Surgically resected colorectal adenocarcinomas showed an increase in both histamine concentration and histidine decarboxylase with increased staging. FAP manifests as intra-abdominal desmoid tumors in 25% of FAP patients^41^. The genetic cause are APC mutations, rather than beta-catenin, so these results may indicate a similar dysregulation of histadine metabolism. The NAD(+) salvage pathway has been identified as important for malignancies, such multiple myeloma^42^ and breast cancer^43^. In three breast cancer cell lines and 176 primary tumor cell lines, Sharif *et al*. found these cell lines had high levels of the rate-limiting enzyme of the NAD(+) salvage pathways, nicotinamide phosphoribosyltransferase (NAMPT) and lower levels of p73, both correlating to poor survival rates. While p53 did not have the same correlation with NAMPT or survival, the p53 pathway was recently identified as regulating Wnt/beta-catenin signaling in mesenchymal progenitor cells^44^. There has been conflicting results in the literature evaluating the role of p53 in desmoid tumor patients. For example, there is data that shows the overexpression of p53 and Ki-67 indicates a high probability of recurrence^45^, while high expression of both beta-catenin and p53 are markers of the risk reduction^46^. Future studies need to evaluate the relationship between NAMPT, p53, and beta-catenin mutations in these mesenchymal tumors.

In addition to the untreated cells, the broad spectrum metabolomics identified differences in the metabolic response to two drugs identified by the ongoing screening efforts. Both Dasatinib and FAK Inhibitor 14 are kinase inhibitors. Dasatinib is l Bcr-Abl and Src family tyrosine kinase inhibitor^14^, while FAK inhibitors target the intersecting integrin and receptor tyrosine kinase signal transduction pathways^15^. Both treatments target proteins that directly interact with beta-catenin upstream of the Wnt pathway. Src and FAK form a dual kinase complex, promote cell motility, cell cycle progression and cell survival^47^. One of their many downstream molecules is Akt, which phosphorylates GSK3B, regulating beta-catenin phosphorylation. It is likely that both inhibitors are affecting these same links, although both Src (and other molecules that Dasatinib potentially inhibits) and FAK have their other independent functions. While FAK activity in desmoid tumors has not yet been evaluated, others have considered Src activity and found it to be variable^48^.

The metabolites that were found to be perturbed in the treated cells were the same as those identified as differentiating the untreated cells. The response to treatment, however, was markedly different by cell and genotype, as expected. The lipid bins were lower in the FAK Inhibitor 14 treated tumor cells, while there were no changes associated with the Dasatinib treatments compared to the vehicle. This was not surprising since tyrosine kinase activity is regulated by lipid membranes and contain a lipid-binding domains^49^. Lipid dysregulation is widely studied in cancer and cancer metabolism as a means of signaling^50,51^; therefore, FAK Inhibitor 14 could constrict the tumorigenesis signaling pathways through the lipids^52^. Additionally, the 1-methylnicotinamide bins were 1.6–3.7-fold higher in the FAK Inhibitor 14 cells compared to vehicle, while no difference was observed in the normal cell lines.

Glucose and lactate were decreased in the FAK Inhibitor 14 treated cells binned data, suggesting an altered energetic state through NAD metabolism. Differences in glucose-1-phosphate and uridine in the T219 treated cells are related to the alterations in energy metabolism. Uridine has been identified as a biomarker in two colorectal cancer studies^53,54^. Nucleotide production is dependent on glycolysis and the TCA cycle metabolic intermediates^55^. Targeting glucose and nucleotide metabolism in cancer research has proved to be difficult but important^56,57^, particularly in the tumor micro-environment^58^.

The two treatments also caused in a difference in the branched chain amino acids (BCAAs). Isoleucine, leucine, and valine have been noted in the literature as markers of cancer as integral players in the mTORC1 pathway^59^. As shown in Supplemental Table 5, the BCAAs all increased, except for the T141 treated with FAK 14 Inhibitor, which decreased 2.3-fold compared to vehicle. Since concentrations of these three amino acids usually trend together.

Asparagine was another amino acid that had an equal but opposite change in response to the treatments. There was a 2.7-fold increase in asparagine for T219 Dasatinib treated cells while decreased in T141 FAK Inhibitor 14 by 2.4-fold. mTORC1 activity and protein synthesis is regulated by asparagine, and helps regulate uptake of serine, arginine and histidine^60^. While these amino acids were not observed as differentiating the cells, the mTORC1 pathway was one of the top pathways that matched the data.

Metabolomics has been used to better understand the impact of another Bcr-Abl tyrosine kinase inhibitor, Gleevec (imatinib), on a gastrointestinal stromal tumor (GIST) T1 cell line^61^. Gleevec has been found to be effective in some desmoid tumor studies^62,63^; therefore, it was expected to identify similar changes in the desmoid study. The most important metabolites for differentiating the treated and untreated GIST cells were decreases in glutathione, glutamate, aspartate, myo-inositol, glycerophosphocholine, and phosphocholine. A decrease in the glutathione, glycerophosphocholine, and phosphocholine were attributed to inhibited growth and invasiveness, while a decrease in the glutamate and glutamine could be indication of a change in glycolysis moving from cytosol to mitochondria. Several differences were observed comparing these metabolites and tyrosine kinase inhibitors. In the desmoid tumor study, aspartate increased in the Dasatinib T219 cells 2.7-fold. No significant myo-inositol differences were observed when the cells were treated in this study. Glutathione metabolism alterations have been previously identified in cervical, colorectal, gastric and esophageal, bone marrow, breast, colon, larynx, and lung cancer^64,65^. FAK 14 Inhibitor caused glutathione to decreased 2-fold in T141 cells, while Dasatinib increased glutathione 1.5-fold in the T219. Glutathione metabolism has been used as a marker of therapeutic efficacy for some time, as it is a marker of proliferative and cell cycle progression^66^. For example, glutathione S-transferase polymorphisms have been evaluated as a chemotherapeutic marker toxicity and reduced survival of childhood acute myeloid leukemia^67^. Future desmoid tumor studies could investigate targeting glutathione S-transferase in desmoid tumor samples to better understand the these differences in therapeutic responses.

While this was a small pilot study, this was the first attempt to utilize the broad spectrum metabolomics to explore matched desmoid tumor and normal fibroblast cell lines in comparison to an unaffected cell line. Differences in the cell lines were found to be related to aminoacyl-tRNA biosynthesis in mitochondria and cytoplasm and signal transduction amino acid-dependent mTORC1 activation pathways. Future studies will investigate if the aminoacyl-tRNA synthetases differentiate these cell lines in follow-up studies, to include aspartyl-tRNA synthetase, glycyl-tRNA synthetase, tyrosyl-tRNA synthetase, and leucyl-tRNA synthetase. Once the pathways and proteins have been verified, there will be a concerted effort to determine the mechanism. Metabolite profile differences were observed in the two beta-catenin genotypes, T41A and S45F. The novel treatments identified by the Desmoid Collaboration for a Cure, Dasatinib and FAK Inhibitor 14, altered the normal and desmoid tumor genotype profiles differentially, signifying a potential opportunity for directed therapies in the future. These results will need to be validated in a much larger data set but provide a tangible step in this rare disease understanding.

## Methods

### Cell lines and culturing

Primary desmoid and normal fibroblast cell cultures were established as previously described^68,69^. Tumor cell line 141 (T141) was genotyped to have the T41A beta-catenin mutation, while tumor cell line 219 (T219) was S45F beta-catenin mutation. Both of the desmoid cell lines had matched normal fibroblast cell lines, which are denoted N141 and N219. For normal fibroblasts, the cells are derived similarly but from skin tissue from the same desmoid tumor patients. Obtaining paired samples is extremely difficult, and it is possible that at the time of this experiment that these matched cell lines are the only ones of their kind. The fibroblast cell line (CRL7518) was purchased from ATCC and culture identically to the primary cell lines; this ATCC cell line served as the unaffected cell line. Monolayer cell cultures were grown in DMEM supplemented with 10% fetal bovine serum and maintained at 37 °C in 5% CO_2_. Cells were divided when confluent and experiments were performed between the third and sixth passages.

### *In vitro* inhibitors treatment and sample preparation

Approximately 10 × 10^6^ cells were treated with 1.0 uM Dasatinib (Selleck, Houston, USA) dissolved in DMSO, or 0.5 µM FAK Inhibitor 14 (Cayman Chemicals Company, Michigan, USA) dissolved in water. Cells were incubated in fresh media containing the inhibitors, or vehicle (DMSO for Dasatinib; H_2_O for FAK Inhibitor 14), for 24 hours. Both Dasatinib and FAK Inhibitor 14 were found to inhibit desmoid cellular growth but not normal fibroblast viability in our on-going screening studies, and there were no visible signs of cell death after the 24 hours. An aliquot of the media following treatment was collected, and the remainder of the media was aspirated. The cells were washed with PBS twice, and quenched with 8 mL ice-cold isotonic 0.9% (w/v) saline for 2 minutes. Total cellular content was then extracted with 1.7 mL ice-cold acetonitrile/water (50:50, v/v) solution. Cell extracts were collected using a cell scraper and quickly transferred to MagNA Lyser Green Beads tubes (Roche, Indianapolis, USA) and stored in −80 °C. Media was added to empty plates and incubated together with the cells for the duration of the experiment served as a blank.

Cells were homogenized on the MagNA Lyzer, with two 30-sec cycles at 2000 rpm, resting in a −20 °C chilling block for 1 min in between pulses, and centrifuged samples at 16,000 rcf for 4 min. The cell lysate was transferred to a new 2 mL Lo-Bind Eppendorf tubes, with the final cell count approximately 10 × 10^6^ cells for each sample. Of the twenty cell lysate samples, six samples had sufficient volume for study samples and to be included in an analytical quality control (QC) total pool. Aliquots from these cell lysate samples were combined, divided into three total pool aliquots, and processed identically to the cell lysate study samples. All study and pool samples were lyophilized to dryness and reconstituted in a 0.2 M phosphate buffer, pH 7.4, in D_2_O with 10% Chenomx ISTD.

### NMR data acquisition and analysis

Data acquisition, statistics, and pathway analysis were performed as previously described^17,70,71^. Three NMR spectra were acquired for each of the individual study samples and the pooled samples. ^1^H NMR spectra of cell lysate samples were acquired on a Bruker Avance III 700 MHz NMR spectrometer (located at the David H. Murdock Research Institute at Kannapolis, NC, USA) using a NOESY1D (noesypr1d) pulse sequence. NMR spectra were pre-processed using ACD 1D NMR Processor 12.0 (ACD Labs, Toronto, Canada). NMR bins (0.50–9.30 ppm) were made after excluding water (4.70–5.20 ppm) and regions with low signal to noise^72^ (5.95–6.85, 8.47–8.85, 9.00–9.25 ppm) using intelligent binning width of 0.04 ppm and 50% looseness factor. Integrals of each of the bins were normalized to total integral of each of the spectrum.

Descriptive statistics and two-sided t-tests, using the Satterthwaite approximation for unequal variances, were conducted for the tumor and normal binned NMR data (SAS Institute Inc, Cary, NC). When there were at least 6 samples in each group of a binary comparison, the Wilcoxon rank sum test was used; for sample sizes smaller than this, the exact Wilcoxon rank was employed. Spectral replicates were treated as independent samples for this pilot study, and p-values < 0.1 were considered to be statistically significant and were not adjusted for multiple testing^73,74^. Normalized binned NMR data were mean centered and Pareto scaled prior to multivariate analysis. Multivariate data analysis methods (e.g. principal component analysis [PCA], orthogonal partial least squares discriminant analysis [OPLS-DA]) were used to reduce the dimensionality and to enable the visualization of the separation of the study groups (SIMCA 14.1, Umetrics, Umeå, Sweden). The PCA plots were inspected to ensure that the pooled samples were tightly clustered in the center of all of the individual study samples, a quality control method that is widely used in metabolites studies^75^. All models used a 7-fold cross-validation to assess the predictive ability of the model (Q^2^). Loadings plots and variable influence on projections (VIP) plots were inspected, and bins that had a VIP ≥ 1.0 with a jack-knife confidence interval that did not include 0 were determined to be important to differentiating the study groups. Chenomx NMR Suite 8.2 Professional software (Edmonton, Alberta, Canada), which has a concentration library of approximately 350 compounds, was used to match the signals in the identified bins to metabolites. Chenomx was also used to semi-quantify metabolites, and all concentrations were adjusted to the cell count for each sample.

Metabolites identified as important (VIP ≥ 1.0, p < 0.1, or magnitude of fold change (FC) > 2) were analyzed for pathway enrichment analysis using the knowledge-based canonical pathways and endogenous metabolic pathways in the MetaCore module in GeneGo software (Chicago, IL). Ranking of relevant pathways was based on hypergeometric p-values.

The metabolomics data are available for download at the NIH Common Fund Metabolomics Data Repository and Coordinating Center at the University of California at San Diego (Dr. Shankar Subramaniam, PI, *U01-DK097430*) under study ST000454.

## Electronic supplementary material

Supplementary Information
