# Supplementary material for: A Metabolomics Pilot Study on Desmoid Tumors and Novel Drug Candidates
Source: Sci Rep. 2018 Jan 12;8:584. doi: 10.1038/s41598-017-18921-7 (PMC5766559; doi:10.1038/s41598-017-18921-7)

A Metabolomics Pilot Study on Desmoid Tumors and Novel Drug Candidates

Kelly A. Mercier [1], Mushriq Al-Jazrawe[2,3], Raymond Poon[2], Zachery Acuff [1], Benjamin Alman[2-4]

Supplemental Table 1. The library matched NMR bins that differentiate the untreated tumor cell lines 141 (T41A) and 219 (S45F) cell lines, where the p values were calculated with the exact Wilcoxon Rank Sum Test (VIP ≥ 1.0, or FC > 2). A positive fold change indicates median of 219 > median of 141.

Library-Matched Metabolite	Chemical Shift	VIP	FC**
Glucose-1-phosphate |Glutamate |Alanine	[3.74 .. 3.80]	4.3	-1.3
Glucose|myo-Inositol | Glycine	[3.51 .. 3.57]	3.2	1.8
Glucose-1-phosphate	[5.42 .. 5.48]	3.0	-1.9
Glucose-1-phosphate | Creatine	[3.89 .. 3.93]	2.9	-1.4
Glutamate | Glutamine	[2.09 .. 2.15]	2.6	-1.2
Glucose-1-phosphate| Serine	[3.83 .. 3.89]	2.6	-1.3
Glutamate | Proline	[2.32 .. 2.37]	2.5	-1.1
Glucose|Glucose-1-phosphate	[3.46 .. 3.51]	2.5	-1.3
myo-Inositol| Threonine	[3.57 .. 3.62]	2.4	1.7
myo-Inositol | Glucose | Taurine	[3.23 .. 3.29]	2.4	1.4
Serine | Unknown	[3.97 .. 4.02]	2.3	1.4
Pyroglutamate | Glutamate | Proline	[2.01 .. 2.07]	2.2	-1.1
myo-Inositol| Choline	[4.02 .. 4.08]	1.9	1.4
Glucose|Glucose-1-phosphate | Taurine	[3.39 .. 3.44]	1.9	-1.2
Glucose-1-phosphate |Glutamate	[3.72 .. 3.74]	1.8	-1.4
Lipids	[0.83 .. 0.89]	1.7	-1.3
Arginine | Lysine	[1.88 .. 1.94]	1.6	1.2
Glutamine	[2.42 .. 2.48]	1.6	-1.3
Alanine| Lipids	[1.44 .. 1.49]	1.5	1.2
Glucose|Glucose-1-phosphate	[3.36 .. 3.39]	1.4	-1.6
Valine	[1.02 .. 1.07]	1.4	1.3
Glutathione	[2.51 .. 2.57]	1.3	1.3
Choline | O-Phosphocholine | O-Acetylcholine | sn-Glycero-3-phosphocholine	[3.19 .. 3.23]	1.3	1.1
Leucine | Lysine| Lipids	[1.68 .. 1.73]	1.2	1.1
Lipids	[1.49 .. 1.55]	1.2	1.2
Phenylalanine	[3.09 .. 3.13]	1.2	1.4

Aspartate	[2.66 .. 2.72]	1.2	1.2
Leucine | Lysine| Lipids	[1.73 .. 1.79]	1.1	1.1
Phenylalanine	[3.13 .. 3.17]	1.1	1.3
Methanol | Proline	[3.33 .. 3.36]	1.1	1.3
Glucose	[4.63 .. 4.68]	1.1	
Creatine | Creatinine | Lysine| Tyrosine	[3.02 .. 3.07]	1.1	1.1
myo-Inositol	[3.29 .. 3.31]	1.0	2.0
Glutathione	[4.54 .. 4.59]	1.0	3.3
Asparagine	[2.83 .. 2.87]	1.0	1.6
Glutathione	[2.57 .. 2.60]	1.0	1.4
Valine | Lipids	[2.24 .. 2.30]	1.0	1.1
Asparagine	[2.92 .. 2.95]	1.0	1.4
Lipids	[1.39 .. 1.44]	1.0	-1.1
Unknown	[4.50 .. 4.54]	0.7	9.9
Lipids	[0.62 .. 0.64]	0.6	2.3
Lipids	[0.57 .. 0.59]	0.6	2.5
1-Methylnicotinamide	[8.95 .. 9.00]	0.5	4.2
p-Methylhistidine	[7.91 .. 7.97]	0.5	4.5
1-Methylnicotinamide	[8.87 .. 8.92]	0.5	4.9
Lipids	[0.52 .. 0.55]	0.5	3.1
1-Methylnicotinamide	[9.27 .. 9.31]	0.4	2.1
Unknown	[7.06 .. 7.08]	0.4	4.8
Unknown	[7.14 .. 7.16]	0.3	6.1

Supplemental Table 2. Semi-quantitated metabolites that were found to be statistically different based on the median values untreated desmoid tumor cell lines 141 (T41A) and 219 (S45F) FC > 2. A positive fold change indicates median of 219 > median of 141.

Semi-Quantified Metabolites	FC**
Asparagine	2.3
Aspartate	2
Glutathione	3.1
Leucine	2.3
Phenylalanine	2.8

Proline	3.4
Tyrosine	2
Valine	2.6

Supplemental Table 3. The library matched NMR bins that differentiate the exposure of Dasatinib compared to DMSO (vehicle) for desmoid, tumor, and unaffected cell lines where the p values were calculated with the Exact Wilcoxon Rank Sum Test (VIP ≥ 1.0, p < 0.1, or FC > 2). No p value was calculated for the unaffected due to the small number of samples.

		Normal			Tumor			Unaffected		
Library-Matched Metabolite	Chemical Shift	VIP	p-value	FC	VIP	p-value	FC	VIP	FC	Exposure Difference
Lipids	[0.57 .. 0.59]	0.6	0.09	-1.3						Normal: DMSO > Das
Lipids	[0.83 .. 0.89]							1.3	-1.1	Unaffected: DMSO > Das
Leucine | Isoleucine	[0.91 .. 0.97]	1.9	0.20	-1.0	1.2	0.94	-1.0	1.9	-1.0	Normal: DMSO > Das; Tumor: DMSO > Das; Unaffected: DMSO > Das
Isoleucine | Valine	[0.97 .. 1.02]	1.3	1.00	-1.0	1.1	0.20	1.1			Normal: DMSO > Das; Tumor: Das > DMSO
Lipids	[1.18 .. 1.23]							1.0	-1.1	Unaffected: DMSO > Das
Isoleucine | 3-Hydroxyisovalerate	[1.23 .. 1.27]				1.0	0.82	-1.1	1.0	-1.1	Tumor: DMSO > Das; Unaffected: DMSO > Das
Isoleucine	[1.27 .. 1.30]	0.6	0.05	1.1						Normal: Das > DMSO
Lactate | Threonine	[1.30 .. 1.34]	3.2	0.20	-1.2	2.7	0.20	-1.4	1.8	-1.1	Normal: DMSO > Das; Tumor: DMSO > Das; Unaffected: DMSO > Das
Lipids	[1.39 .. 1.44]							1.2	-1.1	Unaffected: DMSO > Das
Alanine| Lipids	[1.44 .. 1.49]	1.8	1.00	-1.1						Normal: DMSO > Das
Leucine	[1.66 .. 1.68]	0.9	0.09	-1.1						Normal: DMSO > Das
Leucine | Lysine| Lipids	[1.68 .. 1.73]	1.2	0.59	-1.0				1.4	-1.0	Normal: DMSO > Das; Unaffected: DMSO > Das
Leucine | Lysine| Lipids	[1.73 .. 1.79]	1.0	0.40	-1.0				1.1	-1.0	Normal: DMSO > Das; Unaffected: DMSO > Das
Lipids	[1.79 .. 1.84]							1.2	-1.1	Unaffected: DMSO > Das
Lysine | Arginine	[1.88 .. 1.94]				1.1	0.09	1.1			Tumor: Das > DMSO
Proline	[1.96 .. 1.99]							1.1	-1.1	Unaffected: DMSO > Das
Pyroglutamate | Glutamate | Proline	[2.01 .. 2.07]	1.6	0.94	1.0	1.7	0.49	-1.1	2.1	-1.1	Normal: Das > DMSO; Tumor: DMSO > Das; Unaffected: DMSO > Das
Glutamate | Glutamine	[2.09 .. 2.15]	3.5	0.20	-1.1	3.9	0.12	-1.3	2.8	-1.2	Normal: DMSO > Das; Tumor: DMSO > Das; Unaffected: DMSO > Das
Glutamate | Glutamine | Glutathione	[2.15 .. 2.19]				1.3	1.00	-1.1			Tumor: DMSO > Das
Valine|Lipids	[2.24 .. 2.30]							1.1	-1.1	Unaffected: DMSO > Das
Glutamate	[2.30 .. 2.32]				1.7	0.02	-1.6	1.1	-1.2	Tumor: DMSO > Das; Unaffected: DMSO > Das
Glutamate | Proline	[2.32 .. 2.37]	2.4	0.94	-1.0	2.7	0.82	-1.2	2.7	-1.1	Normal: DMSO > Das; Tumor: DMSO > Das; Unaffected: DMSO > Das
Pyroglutamate | Glutamine	[2.37 .. 2.40]	1.0	0.20	-1.1						Normal: DMSO > Das
Glutamine| Pyroglutamate	[2.40 .. 2.42]	1.4	0.20	-1.2	1.5	0.02	-1.6			Normal: DMSO > Das; Tumor: DMSO > Das
Glutamine	[2.42 .. 2.48]	3.2	0.20	-1.3	3.0	0.70	-1.6			Normal: DMSO > Das; Tumor: DMSO > Das
Glutathione	[2.51 .. 2.57]				1.1	0.70	-1.2			Tumor: DMSO > Das

Glutathione	[2.90 .. 2.92]				1.0	0.023	-1.5			Tumor: DMSO > Das
Asparagine / Glutathione *** In Tumor	[2.92 .. 2.95]	0.7	0.07	-1.1	0.9	0.02	-1.2			Normal: DMSO > Das; Tumor: DMSO > Das
Asparagine	[2.95 .. 2.97]	1.0	0.04	-1.2						Normal: DMSO > Das
Glutathione | Lysine| Lipids	[2.97 .. 3.02]							1.3	-1.1	Unaffected: DMSO > Das
Lysine | Creatine | Creatinine | Tyrosine	[3.02 .. 3.07]	1.4	0.26	-1.0	1.1	1.00	-1.1			Normal: DMSO > Das; Tumor: DMSO > Das
Unknown	[3.07 .. 3.09]				0.9	0.02	-1.5			Tumor: DMSO > Das
Phenylalanine	[3.09 .. 3.13]	1.3	0.26	-1.1	1.1	0.12	-1.2	1.3	-1.2	Normal: DMSO > Das; Tumor: DMSO > Das; Unaffected: DMSO > Das
Phenylalanine	[3.13 .. 3.17]							1.3	-1.2	Unaffected: DMSO > Das
Tyrosine	[3.17 .. 3.19]							1.0	-1.1	Unaffected: DMSO > Das
Choline | O-Phosphocholine | O-Acetylcholine | sn-Glycero-3-phosphocholine	[3.19 .. 3.23]				1.0	1.00	-1.0			Tumor: DMSO > Das
myo-Inositol | Glucose | Taurine	[3.23 .. 3.29]	3.4	0.20	1.2	3.2	0.02	1.4	2.9	1.3	Normal: Das > DMSO; Tumor: Das > DMSO; Unaffected: Das > DMSO
myo-Inositol	[3.29 .. 3.31]	1.2	0.32	1.1						Normal: Das > DMSO
Methanol | Proline	[3.33 .. 3.36]	2.0	0.09	1.2						Normal: Das > DMSO
Glucose|Glucose-1-phosphate	[3.36 .. 3.39]	1.0	1.00	1.2	2.3	1.00	-1.6			Normal: Das > DMSO; Tumor: DMSO > Das
Glucose|Glucose-1-phosphate | Taurine	[3.39 .. 3.44]	3.5	0.20	1.3	4.9	0.02	2.0			Normal: Das > DMSO; Tumor: Das > DMSO
Taurine	[3.44 .. 3.46]							1.1	1.8	Unaffected: Das > DMSO
Glucose|Glucose-1-phosphate	[3.46 .. 3.51]	2.4	1.00	1.3	3.1	0.59	1.5			Normal: Das > DMSO; Tumor: Das > DMSO
Glucose| myo-Inositol | Glycine	[3.51 .. 3.57]	2.5	0.32	1.1	1.2	1.00	-1.0	1.1	1.1	Normal: Das > DMSO; Tumor: DMSO > Das; Unaffected: Das > DMSO
Threonine |myo-Inositol	[3.57 .. 3.62]	1.6	0.70	-1.0	1.3	0.70	-1.1			Normal: DMSO > Das; Tumor: DMSO > Das
Glucose	[3.68 .. 3.70]				0.9	0.03	1.3	1.2	1.4	Tumor: Das > DMSO; Unaffected: Das > DMSO
Glucose	[3.70 .. 3.72]				1.0	1.00	-1.2			Tumor: DMSO > Das
Glucose-1-phosphate |Glutamate	[3.72 .. 3.74]	1.4	1.00	1.1	1.8	1.00	1.1			Normal: Das > DMSO; Tumor: Das > DMSO
Glutamate |Glucose-1-phosphate | Glutathione / Alanine *** for Unaffected	[3.74 .. 3.80]				2.2	0.49	1.1	1.8	-1.1	Tumor: Das > DMSO; Unaffected: DMSO > Das
Glucose-1-phosphate	[3.80 .. 3.83]	1.1	0.49	1.0				1.7	1.2	Normal: Das > DMSO; Unaffected: Das > DMSO
Serine | Glucose-1-phosphate	[3.83 .. 3.89]	2.1	0.94	1.1	2.7	0.70	1.2	1.6	1.1	Normal: Das > DMSO; Tumor: Das > DMSO; Unaffected: Das > DMSO
Glucose-1-phosphate | Creatine	[3.89 .. 3.93]	2.4	0.40	1.1	2.8	0.59	1.3	1.9	1.2	Normal: Das > DMSO; Tumor: Das > DMSO; Unaffected: Das > DMSO
myo-Inositol |Choline	[4.02 .. 4.08]	1.5	1.00	-1.0						Normal: DMSO > Das
Ribose| Lactate	[4.08 .. 4.11]	1.2	0.70	-1.1						Normal: DMSO > Das
Lactate| Proline	[4.11 .. 4.17]							1.7	1.1	Unaffected: Das > DMSO

O-Phosphocholine | Pyroglutamate	[4.17 .. 4.19]	1.1	0.09	1.2						Normal: Das > DMSO
Unknown	[4.19 .. 4.22]				0.8	0.03	-1.1			Tumor: DMSO > Das
Threonine | Uridine	[4.22 .. 4.28]				1.5	0.49	-1.2	1.2	-1.0	Tumor: DMSO > Das; Unaffected: DMSO > Das
Lipids	[4.28 .. 4.34]							1.4	1.1	Unaffected: Das > DMSO
Uridine	[4.34 .. 4.39]	1.8	0.04	1.2				1.5	1.2	Normal: Das > DMSO; Unaffected: Das > DMSO
1-Methylnicotinamide	[4.45 .. 4.50]							1.3	1.1	Unaffected: Das > DMSO
Unknown	[4.50 .. 4.54]							1.1	1.3	Unaffected: Das > DMSO
Glutathione	[4.54 .. 4.59]				1.0	0.02	-1.4	1.2	1.1	Tumor: DMSO > Das; Unaffected: Das > DMSO
Unknown	[4.59 .. 4.63]							1.0	1.2	Unaffected: Das > DMSO
Glucose	[4.63 .. 4.68]							1.4	1.8	Unaffected: Das > DMSO
Glucose	[5.20 .. 5.26]							1.4	3.3	Unaffected: Das > DMSO
Unknown	[5.32 .. 5.37]				0.6	0.05	2.4	1.1	3.1	Tumor: Das > DMSO; Unaffected: Das > DMSO
Unknown	[5.37 .. 5.42]							1.3	15.1	Unaffected: Das > DMSO
Glucose-1-phosphate	[5.42 .. 5.48]	1.9	1.00	1.2	2.6	0.70	1.6	1.0	41.6	Normal: Das > DMSO; Tumor: Das > DMSO; Unaffected: Das > DMSO
Unknown	[5.59 .. 5.65]	1.2	0.49	-1.2						Normal: DMSO > Das
Uridine	[5.88 .. 5.94]				0.7	0.09	1.3	1.5		Tumor: Das > DMSO; Unaffected: Das > DMSO
Uridine	[7.84 .. 7.89]							1.2	5.2	Unaffected: Das > DMSO
Unknown	[8.33 .. 8.38]				0.7	0.29	-2.0			Tumor: DMSO > Das
Unknown	[8.44 .. 8.47]	0.4	0.32	2.1				0.6	-2.1	Normal: Das > DMSO; Unaffected: DMSO > Das

Supplemental Table 4. The library matched NMR bins that differentiate the exposure of FAK Inhibitor 14 compared to H_2O (vehicle) for desmoid, tumor, and unaffected cell lines where the p values were calculated with the Exact Wilcoxon Rank Sum Test (VIP ≥ 1.0, p < 0.1, or FC > 2). No p value was calculated for the unaffected due to the small number of samples.

		Normal			Tumor			Unaffected		
Library-Matched Metabolite	Chemical Shift	VIP	p-value	FC	VIP	p-value	FC	VIP	FC	Exposure Difference
Lipids	[0.68 .. 0.70]	0.6	0.05	1.1						Normal: FAK > H_2O
Lipids	[0.74 .. 0.77]	0.7	0.04	1.1						Normal: FAK > H_2O
Lipids	[0.77 .. 0.79]	0.5	0.09	1.1						Normal: FAK > H_2O
Lipids	[0.83 .. 0.89]				1.3	0.32	-1.1	2.1	-1.1	Tumor: H_2O > FAK; Unaffected: H_2O > FAK
Lipids|Glycocholate	[0.89 .. 0.91]	1.1	0.07	1.1				1.2	-1.1	Normal: FAK > H_2O; Unaffected: H_2O > FAK
Leucine | Isoleucine	[0.91 .. 0.97]							1.5	1.0	Unaffected: FAK > H_2O
Valine | Isoleucine	[0.97 .. 1.02]							3.4	1.2	Unaffected: FAK > H_2O
Valine	[1.02 .. 1.07]							2.0	1.1	Unaffected: FAK > H_2O
Lipids	[1.18 .. 1.23]							1.2	-1.0	Unaffected: H_2O > FAK
Isoleucine	[1.27 .. 1.30]							1.2	-1.1	Unaffected: H_2O > FAK
Threonine | Lactate	[1.30 .. 1.34]							1.4	1.0	Unaffected: FAK > H_2O
Lipids	[1.34 .. 1.36]	0.7	0.05	1.1						Normal: FAK > H_2O
Lipids	[1.36 .. 1.39]	0.9	0.04	1.1						Normal: FAK > H_2O
Lipids	[1.39 .. 1.44]				1.0	0.32	-1.1	1.6	-1.1	Tumor: H_2O > FAK; Unaffected: H_2O > FAK
Alanine| Lipids	[1.44 .. 1.49]							1.8	1.0	Unaffected: FAK > H_2O
Lipids	[1.49 .. 1.55]							1.1	-1.0	Unaffected: H_2O > FAK
Lipids	[1.55 .. 1.57]	0.8	0.02	1.1						Normal: FAK > H_2O
Lipids	[1.57 .. 1.59]	0.6	0.07	1.1						Normal: FAK > H_2O
Lipids	[1.59 .. 1.61]	0.7	0.09	1.1						Normal: FAK > H_2O
Leucine	[1.61 .. 1.64]	1.0	0.02	1.1						Normal: FAK > H_2O
Leucine	[1.64 .. 1.66]	1.0	0.02	1.1						Normal: FAK > H_2O
Leucine	[1.66 .. 1.68]	1.1	0.02	1.1						Normal: FAK > H_2O
Leucine | Lysine| Lipids	[1.68 .. 1.73]							1.2	-1.0	Unaffected: H_2O > FAK
Leucine | Lysine| Lipids	[1.73 .. 1.79]				1.2	0.49	-1.0			Tumor: H_2O > FAK

Lipids	[1.79 .. 1.84]							1.1	-1.0	Unaffected: H_2O > FAK
Lysine	[1.84 .. 1.86]	1.1	0.02	1.2						Normal: FAK > H_2O
Lysine| Lipids	[1.86 .. 1.88]	0.7	0.09	1.1						Normal: FAK > H_2O
Lysine| Proline	[1.94 .. 1.96]	0.8	0.07	1.1						Normal: FAK > H_2O
Proline	[1.96 .. 1.99]							1.1	1.0	Unaffected: FAK > H_2O
Glutamate|Proline|Pyroglutamate	[2.01 .. 2.07]	2.3	0.12	-1.1	2.4	0.20	-1.0			Normal: H_2O > FAK; Tumor: H_2O > FAK
Glutamate | Glutamine	[2.09 .. 2.15]	3.7	0.02	-1.1				2.3	1.0	Normal: H_2O > FAK; Unaffected: FAK > H_2O
Glutamate | Glutamine | Glutathione	[2.15 .. 2.19]				2.1	0.02	-1.2	1.9	-1.1	Tumor: H_2O > FAK; Unaffected: H_2O > FAK
Glutamate| Proline	[2.32 .. 2.37]	3.0	0.20	-1.1						Normal: H_2O > FAK
Pyroglutamate | Glutamine	[2.37 .. 2.40]	0.9	0.05	-1.1						Normal: H_2O > FAK
Glutamine| Pyroglutamate	[2.40 .. 2.42]				1.1	0.05	1.1			Tumor: FAK > H_2O
Glutamine	[2.42 .. 2.48]	2.1	0.32	-1.2				1.4	1.0	Normal: H_2O > FAK; Unaffected: FAK > H_2O
Glutathione	[2.51 .. 2.57]							1.6	-1.1	Unaffected: H2O > FAK
Methionine	[2.62 .. 2.66]	1.3	0.02	-1.1				1.7	1.2	Normal: H_2O > FAK; Unaffected: FAK > H_2O
Aspartate	[2.66 .. 2.72]	1.1	0.05	-1.1				1.0	-1.0	Normal: H_2O > FAK; Unaffected: H_2O > FAK
Aspartate	[2.77 .. 2.80]	0.7	0.09	-1.1	1.0	0.16	1.2			Normal: H_2O > FAK; Tumor: FAK > H_2O
Asparagine	[2.92 .. 2.95]				1.1	0.09	-1.3	1.0	-1.1	Tumor: H_2O > FAK; Unaffected: H_2O > FAK
Asparagine	[2.95 .. 2.97]				1.1	0.09	-1.3	1.2	-1.1	Tumor: H_2O > FAK; Unaffected: H_2O > FAK
Glutathione | Lysine| Lipids	[2.97 .. 3.02]							1.1	-1.0	Unaffected: H_2O > FAK
Phenylalanine	[3.09 .. 3.13]				1.3	0.26	-1.2			Tumor: H_2O > FAK
Choline | O-Acetylcholine | O-Phosphocholine | sn-Glycero-3-phosphocholine	[3.19 .. 3.23]				3.2	0.02	-1.3	1.5	-1.0	Tumor: H_2O > FAK; Unaffected: H_2O > FAK
Glucose | Taurine | myo-Inositol	[3.23 .. 3.29]	2.4	0.20	-1.0	3.4	0.20	-1.3			Normal: H_2O > FAK; Tumor: H_2O > FAK
myo-Inositol	[3.29 .. 3.31]	1.5	0.20	-1.3						Normal: H_2O > FAK
Proline	[3.31 .. 3.33]				1.0	0.49	1.2			Tumor: FAK > H_2O
Methanol | Proline	[3.33 .. 3.36]				1.2	0.32	-1.1	1.9	-1.2	Tumor: H_2O > FAK; Unaffected: H_2O > FAK
Glucose| Glucose-1-phosphate	[3.36 .. 3.39]	1.3	0.32	1.1						Normal: FAK > H2O
Glucose| Glucose-1-phosphate | Taurine	[3.39 .. 3.44]	2.9	0.09	1.1	3.6	0.12	-1.4	2.0	1.1	Normal: FAK > H_2O; Tumor: H_2O > FAK; Unaffected: FAK > H_2O
Taurine	[3.44 .. 3.46]							1.2	1.2	Unaffected: FAK > H_2O

Glucose-1-phosphate	[3.46 .. 3.51]							2.3	1.2	Unaffected: FAK > H_2O
Glucose | myo-Inositol | Glycine	[3.51 .. 3.57]	4.0	0.26	-1.2				2.1	1.1	Normal: H_2O > FAK; Unaffected: FAK > H_2O
Threonine | Valine | myo-Inositol	[3.57 .. 3.62]							1.1	1.0	Unaffected: FAK > H_2O
Isoleucine | Unknown	[3.62 .. 3.68]							2.4	1.1	Unaffected: FAK > H_2O
Leucine | Unknown	[3.70 .. 3.72]							1.5	1.1	Unaffected: FAK > H_2O
Leucine	[3.72 .. 3.74]							1.7	1.1	Unaffected: FAK > H_2O
Glucose-1-phosphate |Glutamate |Alanine	[3.74 .. 3.80]							1.2	-1.0	Unaffected: H_2O > FAK
Glucose-1-phosphate	[3.80 .. 3.83]							1.8	1.1	Unaffected: FAK > H2O
Serine| Glucose-1-phosphate	[3.83 .. 3.89]	3.2	1.00	1.1	3.1	0.32	1.1	2.0	1.0	Normal: FAK > H_2O; Tumor: FAK > H_2O; Unaffected: FAK > H_2O
Serine | Unknown	[3.93 .. 3.97]	1.3	0.20	1.1				1.1	1.0	Normal: FAK > H_2O; Unaffected: FAK > H_2O
myo-Inositol |Choline	[4.02 .. 4.08]	2.5	0.32	-1.2				1.4	1.0	Normal: H_2O > FAK; Unaffected: FAK > H_2O
Lactate | Unknown	[4.08 .. 4.11]							1.6	1.1	Unaffected: FAK > H_2O
Lactate| Proline	[4.11 .. 4.17]				1.4	0.12	-1.1	1.7	-1.0	Tumor: H_2O > FAK; Unaffected: H_2O > FAK
Unknown	[4.19 .. 4.22]	0.7	0.09	-1.1						Normal: H_2O > FAK
Threonine | Uridine	[4.22 .. 4.28]							1.9	-1.1	Unaffected: H_2O > FAK
Lipids	[4.28 .. 4.34]							2.0	-1.1	Unaffected: H_2O > FAK
Uridine	[4.34 .. 4.39]							1.5	-1.1	Unaffected: H_2O > FAK
1-Methylnicotinamide	[4.45 .. 4.50]							1.4	-1.1	Unaffected: H_2O > FAK
Unknown	[4.50 .. 4.54]							1.1	-1.3	Unaffected: H_2O > FAK
Glutathione	[4.54 .. 4.59]							1.3	-1.2	Unaffected: H_2O > FAK
Unknown	[5.37 .. 5.42]	0.9	0.32	2.2	0.2	0.32	2.3			Normal: FAK > H_2O; Tumor: FAK > H_2O
Glucose-1-phosphate	[5.42 .. 5.48]	2.5	0.40	1.1						Normal: FAK > H_2O
Unknown	[5.48 .. 5.53]				0.2	0.17	6.0			Tumor: FAK > H_2O
Unknown	[5.59 .. 5.65]	1.2	0.02	-1.3				1.4	1.2	Normal: H_2O > FAK; Unaffected: FAK > H_2O
Uracil	[5.76 .. 5.82]	0.4	0.62	-5.9						Normal: H_2O > FAK
Unknown	[7.57 .. 7.62]				0.3	0.12	3.4			Tumor: FAK > H_2O
Unknown	[7.71 .. 7.76]				0.3	0.20	5.4			Tumor: FAK > H_2O
Adenine	[8.17 .. 8.22]	0.9	0.04	-1.1						Normal: H_2O > FAK
Unknown	[8.22 .. 8.28]				0.3	0.12	2.6			Tumor: FAK > H_2O

Unknown	[8.33 .. 8.38]				0.2	0.390	2.2	Tumor: FAK > H_2O
Unknown	[8.44 .. 8.47]	0.5	0.20	-2.5	0.2	0.24	11.5	Normal: H_2O > FAK; Tumor: FAK > H_2O
1-Methylnicotinamide	[8.87 .. 8.92]				0.2	0.09	1.6	Tumor: FAK > H_2O
1-Methylnicotinamide	[8.92 .. 8.95]				0.1	0.19	3.7	Tumor: FAK > H_2O

Supplemental Table 5. Semi-quantitated metabolites that were found to be statistically different based on the median values for normal and desmoid tumor cell lines 141 (T41A) and 219 (S45F) where the positive fold change indicates median Dasatinib/FAK Inhibitor 14 vs vehicle. The p value was calculated for the normal cells with Wilcoxon Rank Sum Test. No p value was calculated for the individual tumor cell lines due to the small number of samples.

	Dasatinib				FAK Inhibitor 14			
	Normal		T141	T219	Normal		T141	T219
Metabolite	p-value	FC	FC	FC	p-value	FC	FC	FC
Asparagine				2.7			-2.4	
Aspartate				1.8				
Dimethylamine				1.9	0.017	-1.8	-4	
Glucose-1-phosphate				25.5	0.093	-1.3		
Glutamate				1.4			-2.1	
Glutamine					0.017	-1.8		2
Glutathione	0.023	-1.6		1.5			-2	
Isoleucine				1.5			-2.3	2.6
Leucine				1.9				
Phenylalanine					0.093	-1.5		
Proline				2.7			-2.1	
Pyroglutamate	0.054	-2			0.054	-1.8		2.1
Uridine				1.7				-8.8
Valine				1.9				2.3

Supplemental Figures

Figure 1. The supervised multivariate analysis (OPLS-DA) of the 141 (T41A) and 219 (S45F) desmoid tumor cell lines [1 predictive + 1 orthogonal components, R2X =0.93, R2Y =0.96, Q2 (cum) =0.94].

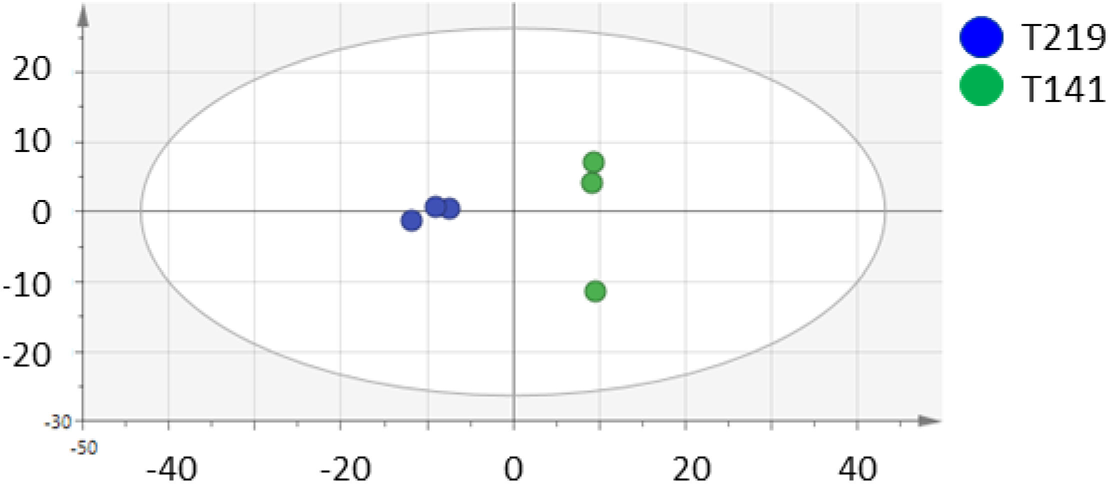

Figure 2. The supervised multivariate analysis (OPLS-DA) of the normal, tumor, and unaffected cell lines when treated with Dasatinib vs DMSO (A-C) and FAK Inhibitor 14 vs H_2O (D-F). A. Normal cells with Dasatinib and DMSO [1 predictive + 1 orthogonal components, R2X =0.89, R2Y =0.82, Q2 (cum) =0.64]. B. Desmoid tumor cells with Dasatinib and DMSO [1 predictive + 2 orthogonal components, R2X =0.95, R2Y =1.0, Q2 (cum) =0.98]. C. Unaffected cells with Dasatinib and DMSO [1 predictive + 1 orthogonal components, R2X =0.98, R2Y =1.0, Q2 (cum) =0.98]. D. Normal cells with FAK Inhibitor 14 vs H_2O [1 predictive + 1 orthogonal components, R2X =0.39, R2Y =0.98, Q2 (cum) =0.87]. E. Desmoid tumor cells with Dasatinib is l Bcr-Abl and Src family tyrosine kinase inhibitor,12 while FAK inhibitors target the intersecting integrin and receptor tyrosine kinase signal transduction pathways.13 [1 predictive + 1 orthogonal components, R2X =0.45, R2Y =0.85, Q2 (cum) =0.69]. F. Unaffected cells with FAK Inhibitor 14 vs H_2O [1 predictive + 1 orthogonal components, R2X =0.87, R2Y =1.0, Q2 (cum) =1.0].

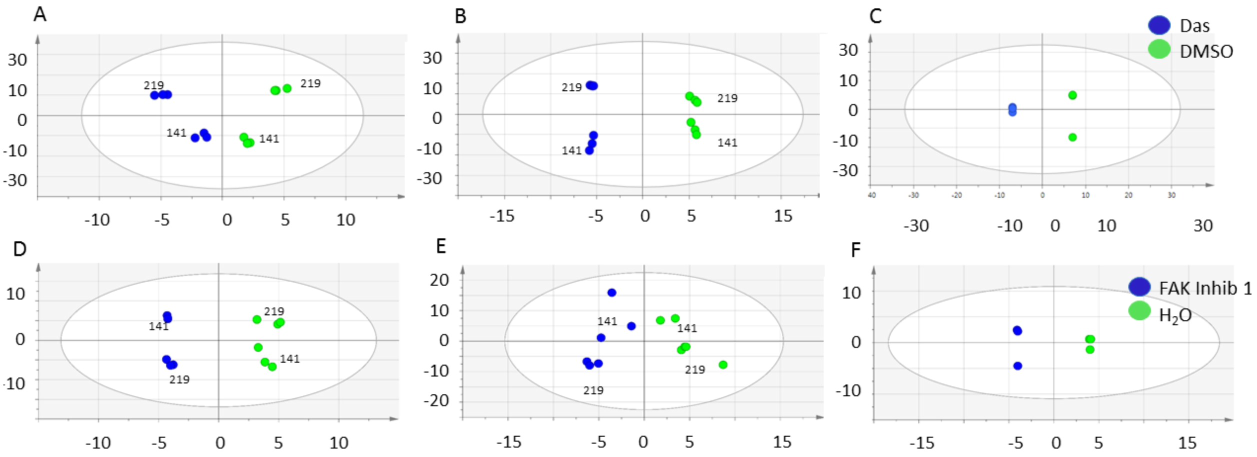

Supplement: Supplementary file 1 — Supplementary Information [file 41598_2017_18921_MOESM1_ESM.pdf]
